# Is brain perfusion correlated to switching mood states and cognitive impairment in bipolar disorder type I? A longitudinal study using perfusion imaging approach

**DOI:** 10.3389/fpsyt.2023.1244134

**Published:** 2023-10-04

**Authors:** Maria Anayali Estudillo-Guerra, Clas Linnman, Victor Galvez, Gina Chapa-Koloffon, Kevin Pacheco-Barrios, Leon Morales-Quezada, Monica Flores Ramos

**Affiliations:** ^1^Clínica de Trastornos del Afecto, Instituto Nacional de Psiquiatría “Ramón de la Fuente”, Mexico City, Mexico; ^2^Spaulding Rehabilitation Hospital and Massachusetts General Hospital, Harvard Medical School, Boston, MA, United States; ^3^Spaulding Neuroimaging Laboratory, Spaulding Rehabilitation Hospital and Massachusetts General Hospital, Boston, MA, United States; ^4^Laboratorio de Neurociencias Cognitivas y Desarrollo, Escuela de Psicología, Universidad Panamericana, Mexico City, Mexico; ^5^Hospital Infantil de Mexico Federico Gómez, Mexico City, Mexico; ^6^Neuromodulation Center and Center for Clinical Research Learning, Spaulding Rehabilitation Hospital and Massachusetts General Hospital, Harvard Medical School, Boston, MA, United States; ^7^Vicerrectorado de Investigación, Unidad de Investigación Para la Generación y Síntesis de Evidencias en Salud, Universidad San Ignacio de Loyola, Lima, Peru; ^8^Subdirección de Investigaciones Clínicas, Instituto Nacional de Psiquiatría Ramón de la Fuente Muñiz, Mexico City, Mexico; ^9^Facultad de Medicina, Universidad Nacional Autónoma de México, Mexico City, Mexico

**Keywords:** bipolar disorder, SPECT, brain imaging, cognitive function, neuroimaging, nuclear medicine

## Abstract

Type I Bipolar disorder (BD-I) is a neuropsychiatric disorder characterized by manic or mixed-featured episodes, impaired cognitive functioning, and persistent work and social functioning impairment. This study aimed to investigate within-subject; (i) differences in brain perfusion using Single-photon emission computed tomography (SPECT) between manic and euthymic states in BD-I patients; (ii) explore potential associations between altered brain perfusion and cognitive status; and (iii) examine the relationship between cerebral perfusion and mania symptom ratings. Seventeen adult patients diagnosed with BD-I in a manic episode were recruited, and clinical assessments, cognitive tests, and brain perfusion studies were conducted at baseline (mania state) and a follow-up visit 6 months later. The results showed cognitive impairment during the manic episode, which persisted during the euthymic state at follow-up. However, no significant changes in brain perfusion were observed between the manic and euthymic states. During mania, trends toward decreased perfusion in the left cerebellum and right superior parietal lobule were noted. Additionally, trends indicated a higher perfusion imbalance in the left superior and middle frontal gyrus during mania and the right superior and middle frontal gyrus during euthymia. No significant correlations existed between brain perfusion, mania symptom ratings, and cognitive performance, indicating that symptomatology might represent more than neural hemodynamics. These findings suggest that cognitive impairment may persist in BD-I patients and highlight the need for therapeutic interventions targeting cognitive deficits. More extensive studies with extended follow-up periods are warranted further to investigate brain perfusion and cognitive functioning in BD-I patients.

## 1. Introduction

Type I Bipolar disorder (BD-I) is a common neuropsychiatric disorder with a lifetime worldwide prevalence of approximately 1% (1). It is characterized by at least one lifetime manic or mixed-featured episode, and it may be accompanied by impaired attentional processing, executive function, verbal memory, and persistently impaired work and social functioning (2, 3); these deficits can be observed in all stages, including euthymia (phase of normality between episodes of mania or depression) (4–6). Brain lesions evidence shows that mania occurs in up to 30% of BD-I patients with basal ganglia calcification, associated with right-sided destructive lesions and with the left-sided epileptogenic lesion, deriving a laterality imbalance (7). However, functional neuroimaging studies have found previous alterations in limbic structures and prefrontal areas, possibly related to cognitive impairment (8). Nonetheless, the current evidence to determine this relationship is inconclusive because it is based chiefly on cross-sectional designs in various patient groups. This approach limits comparison (and correlations) between the different clinical states due to inter-subject differences (9).

Longitudinal studies investigating subjects during manic, euthymic, and depressed episodes promise to capture disease-specific within-subject alterations, as the switch between mood states is a hallmark of BD-I patients. Such designs are challenging, and only a few studies show images of subjects across mood episodes (10–14), and these studies used Magnetic Resonance Imaging to study functional activation and connectivity changes. Cerebral blood flow (CBF) abnormalities have been previously described in patients with Major Depressive Disorder (MDD) and Schizophrenia (15–17). A systematic review of 33 studies compared CBF findings in BD and healthy control subjects (HC) at rest and in response to cognitive and emotional tasks; the most consistent finding was reduced CBF in BD in the cingulate gyrus, frontal, and anterior temporal regions during either depressive or manic stages, compared to healthy controls. However, longitudinal measures of CBF across mood states are rare: Most relevant to the present study, in longitudinal studies contrasting symptomatic (mania or depression) with euthymia, a right–left asymmetry in anterior temporal lobes was observed in the pathological mood states (18) A review of perfusion-weighted magnetic resonance imaging studies in BD found results that supported the presence of hyper-perfusion in the cingulate cortex and frontotemporal regions, as well as the company of hypo-perfusion in the cerebellum in BD subjects when compared with HC and subjects with unipolar depression (19). A study of perfusion fluctuation and perfusion connectivity in BD subjects measured by dynamic arterial spin labeling found that BD subjects exhibited significantly increased perfusion fluctuations in the left fusiform and inferior temporal regions and marginally increased perfusion fluctuations in the right temporal pole and inferior temporal areas, and increased perfusion connectivity between anterior cingulate cortex and the occipitoparietal cortex. Positive symptoms were negatively associated with anterior cingulate cortex perfusion connectivity to the right orbitofrontal and superior frontal regions and right orbitofrontal and inferior frontal regions (20).

Regarding possible changes in brain perfusion, as they relate to genitive function, prior results have been mixed: Regarding CBF in relation to cognitive and emotional tasks comparing BD and HC subjects, it was found that decreased CBF in BD group during memory tasks, increased CBF in prefrontal and limbic regions in BD group and parietal and premotor areas of HC group during serial reaction time tasks, decreased CBF in the dorsolateral prefrontal cortex in BD group during verbal learning tasks, as well as increased CBF in dorsal anterior cingulate cortex regions and decreased CBF in left frontal pole in BD group during decision-making tasks. No differences were found between the groups in studies that used color-word inhibition and verbal fluency tasks. In studies without a HC group, a correlation was found between worse performance on memory and verbal learning and low frontal CBF; also, the psychomotor performance was related to greater anterior temporal CBF in baseline CBF and subsequent cognitive performance with increased CBF in left inferior opercular frontal gyrus in a before and after 4-week cognitive training study. Correlations between CBF and cognitive performance were reported, noting that lower CBF was associated with poorer performance on measured memory tasks, verbal learning, response inhibition, and complex abstraction.

In a previous study, we began exploring cognitive status and brain perfusion (measured by SPECT) during a manic episode in 10 patients with BD-, reporting a positive association between cognitive functioning impairments (verbal learning, verbal fluency, and processing speed) with perfusion in the right temporal pole and a negative association with perfusion in the orbitofrontal cortex and subgenual cingulate cortex, from right hemisphere (21). We expand on these results using a larger sample size, longitudinal design, and quantitative voxel-wise neuroimaging analysis.

The present study aimed to describe within-subject differences in brain perfusion between mania and euthymia; specifically, we explored if the switch from mania to euthymia incurred changes in the laterality of perfusion, with the hypothesis that the mania state would be associated with a higher imbalance in perfusion, favoring relatively higher perfusion of the left cerebrum, based on prior neuroimaging reports (7, 22). Additionally, we sought to explore potential associations between altered brain perfusion reductions in CBF in cingulate, frontal, and anterior temporal regions, as per the prior literature and cognitive status, capitalizing on the within-subject design. Lastly, we characterized the relationship between cerebral perfusion and mania symptom ratings in the whole sample.

## 2. Methods

### 2.1. Subjects

Between March 2015 and March 2019, we recruited 17 adult patients diagnosed with BD-I undergoing a moderate or severe manic episode according to the Young Mania Scale (YMRS; YMRS score ≥ 20) (23, 24), in the National Institute of Psychiatry Ramón de la Fuente Muñiz (INPRFM). Participants were diagnosed according to the DSM IV-TR criteria (25) by an experienced psychiatrist using the South and Central America version of the International Neuropsychiatric Interview (MINI) (26). We included participants with BD-I diagnoses of no longer than 5 years, without current pharmacological treatment, and with no history of electroconvulsive therapy for at least 6 months before the initial evaluation. Patients with a score ≥ 19 on the Montgomery-Asberg Depression Scale for Depression (MADRS) (27), with neuropsychiatric comorbidities, uncontrolled medical conditions, alcohol or other substance use, as well as pregnant or lactating women, were excluded. This study was approved by the Institutional Ethical Review Board of the National Institute of Psychiatry “Ramón de la Fuente Muñiz.” According to the Institution’s guidelines, all participants or legal representatives received a study explanation and signed informed consent before entering the study.

### 2.2. Clinical and cognitive assessments

A complete medical history, a physical examination, a hematological biochemical evaluation (blood biometry, blood chemistry, liver function, and thyroid function), a general urine examination, and an electrocardiogram were obtained for each participant. Regarding cognitive functioning, we assessed immediate verbal learning, fluency, and processing speed. Ten subjects were evaluated using the Immediate Verbal Learning Test (VLT-I), the Verbal Fluency Test (VFT), and the Processing Speed Test (PST) subtests of the Cognitive Impairment in Psychiatry (SCIP-S) Screen Scale Spanish version (28). Seven subjects were assessed using the Hopkins Verbal Learning Test-Revised (HVLT-R) (29) to assess immediate verbal learning, the animal categorical fluency test to assess verbal fluency, and the Brief Assessment of Cognition in Schizophrenia-Symbol Coding test (BACS-SC) (30) to assess processing speed. These assessments were performed at baseline (mania state) and follow-up visits 6 months later. The test scores were normalized and standardized according to each instrument’s cut points using the following formula: *a*/*b* = *c*/*x*.

### 2.3. Neuroimaging protocol

Perfusion studies were performed on participants in a manic state at the INPRFM. The protocol was performed during the resting state using two-head SPECT–CT (PRECEDENCE-Philips). A radiopharmaceutical 925 MBq of Tc99m-ethyl cysteine iReimer (Neurolite R Accesofarm) was administered for 40–45 min.

### 2.4. Statistical analysis

For the descriptive analysis of categorical variables, absolute and relative frequencies were obtained. For quantitative variables, means, medians, and their respective dispersion measures were calculated. The normality of the distribution was evaluated graphically and through the Shapiro–Wilk test. The Spearman rank correlation (correlation between cognitive domains and clinical variables) was performed. A *p* < 0.05 was considered statistically significant with a 95% confidence interval. Due to the exploratory nature of this analysis, we did not correct by multiple comparisons to avoid the type II error. The analysis was performed in the statistical software Stata (version 15.0).

The brain imaging data were modeled in SPM 12 using a multiple linear regression approach. Individual subjects’ mania- and euthymia—SPECT perfusion images were co-registered to compute an average image. This average was normalized to MNI space, and transformations were applied to the mania- and euthymia images, thus avoiding an order bias in co-registration. The spatially normalized images were further smoothed with a 16 mm full-width-half-max filter. The preprocessed images were entered in a repeated measures *t*-test model, controlling for scan global intensities using an ANCOVA regressor.

To analyze laterality effects (the main aim of this study), the raw mania and euthymia-perfusion images were right–left flipped, co-registered to the non-flipped average, and preprocessed as above. Changes in the laterality of perfusion between mania and euthymia were determined by contrasting non-flipped and converted perfusion images in mania versus those in euthymia. Lastly, we utilized the entire sample of subjects evaluated in the mania state to assess the potential correlations between brain perfusion and cognitive outcomes, as well as with symptoms, as rated on the YMRS scale.

For all analyses, the cluster forming threshold was set at *p* < 0.001, and significance was set at *p* < 0.05, corrected by family-wise error rate. Trends for clusters with more than five contiguous voxels at *p* < 0.001, not surviving correction for multiple comparisons, are also reported.

In addition to the above analysis, we further explored results using threshold-free cluster enhancement (TFCE), an approach introduced to increase the sensitivity of voxel-based analyses applying 5,000 permutations and optimizing voxel-level thresholding (31), and by defining regions of interest based on prior literature in the cingulate, frontal lobe, and the temporal poles, determined using the WFU. pickatlas tool (32) and the A.A.L. library (33).

## 3. Results

### 3.1. Participants and ratings

We included 17 patients in the study, 14 women and three men. The mean age was 41.2 (SD = 15. 09; Table 1). The cognitive domains of immediate verbal learning, verbal fluency, and processing speed demonstrated performance below the typical threshold at baseline (Table 2). Additionally, there was no observed correlation between YMRS scores and cognitive functioning.

**Table 1:** Clinical and sociodemographic characteristics.

Variable		*n*	%
Sex	Women	14	82.3
	Men	3	17.6
		Mean	Min-Max
Age		37	20–67
Years of schooling		13.47	5–19
Duration of the last manic episode (weeks)		4.02	1–16
Time since diagnosis (years)		2.5	0–4
Number of previous episodes of mania		1.29	1–3
Number of prior episodes of depression		1.11	0–4

**Table 2:** Clinical and cognitive functioning.

SCALES	The score during the episode of Mania *n* = 17	Score on 6-month follow-up *n* = 8	Interpretation of the results
	Stocking (min-max)	Stocking (min-max)	
MADRS	5.17 (0–14)	5.82 (0–11)	≥ 35: Severe depression
			20–34: Moderate depression
			7–19: Mild depression
			≤ 6: Depression in recovery
YMRS	32.82 (20–56)	1.87 (0–4)	≤6: Euthymia
			7–20: Mixed episode
			>20: Manic episode
BPRS	32.17 (20–47)	25.12 (21–28)	0–9: Absence of the disorder.
			10–20: Mild disorder
			≥21: Severe disorder
			Normal values^*^
Immediate verbal learning	18.32 (7–27)	17.12 (9–25)	<21
Verbal fluency	16.35 (6–28)	19.12 (12–27)	<19
Processing speed	9.95 (5–15.8)	9.62 (5–14)	<12

MADRS, Montgomery-Asberg Depression Rating Scale; YMRS, Young Mania Rating Scale; BPRS, Brief Psychiatry Rating Scale; SCIP-I, screen for cognitive impairment in psychiatry. ^*^Reference values are taken from Rojo et al. (34).

During the follow-up, 6 months later, eight out of the 17 patients were evaluated (nine participants discontinued their participation due to personal reasons and time availability). An expected significant difference between baseline and follow-up measurements was found in YMRS scores (*p* < 0.001). No differences were found between the cognitive outcomes and the rest of the clinical assessments, even though all subjects were euthymic, and none of them were depressed according to the MADRS scores. As no significant changes in cognition were discerned between the mania and the euthymia, we did not pursue the planned correlation analyses of changes in cognition about changes in perfusion. Pharmacological treatment a follow-up are described in Supplementary Table 1.

**Figure 1:**
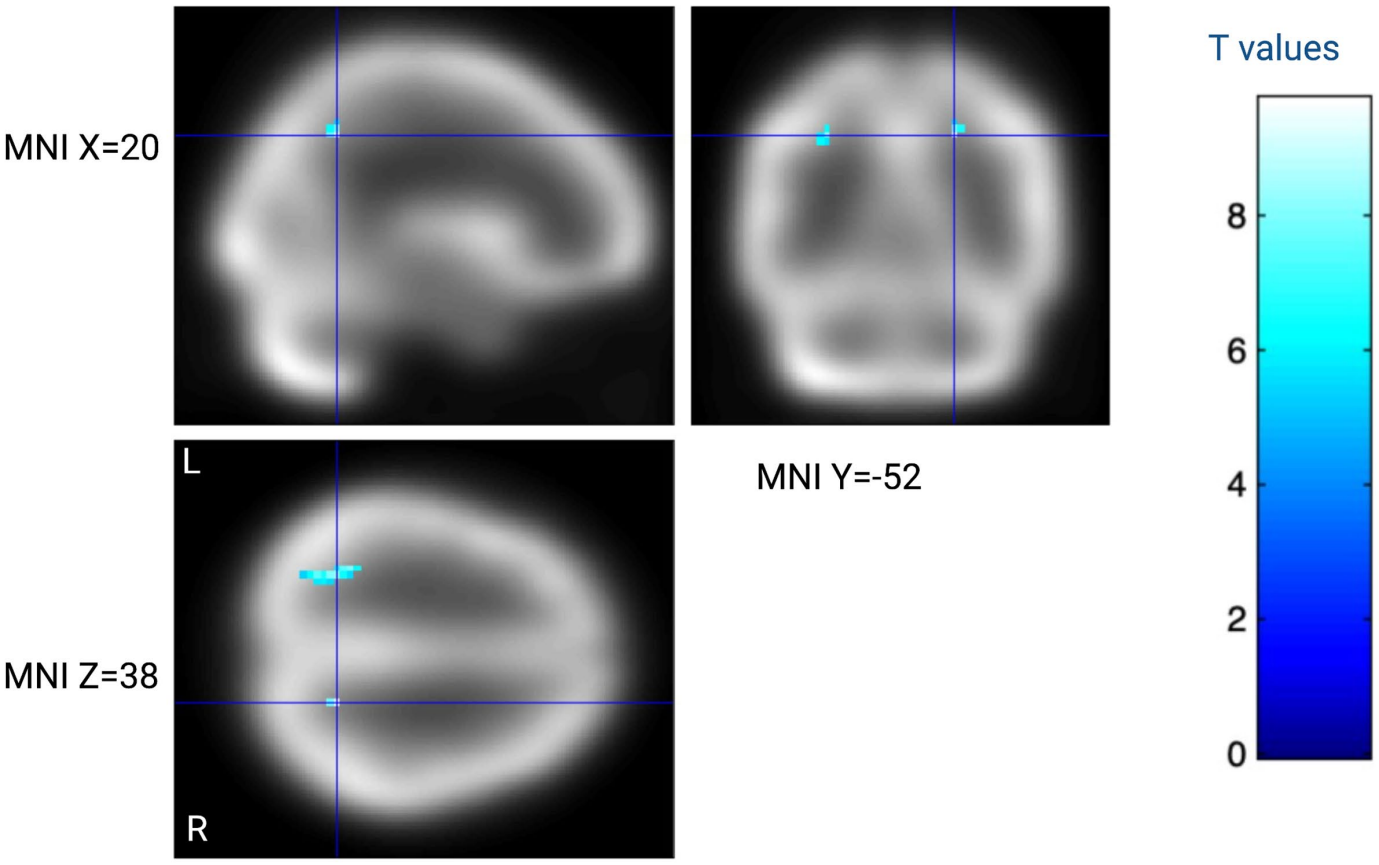
Within-subject analysis contrasting eight subjects with BD-1 assessed during mania and at 6 month follow-up. Blue regions indicate lower cerebral perfusion during mania as compared to euthymia at T > 5.2 (*p* < 0.001 not corrected for multiple comparisons). The color bar indicates within-subject *t*-test *t*-values. Created with BioRender.com.

Brain perfusion was not significantly different between the mania and the euthymia state. However, at a less stringent threshold (*p* < 0.001, not corrected for multiple comparisons), a trend toward decreased perfusion in the mania state was observed in the left cerebellum and the right superior parietal lobule, see Table 3 and Figure 1.

**Table 3:** Brain perfusion results.

Analysis	Contrast	Cluster size	T	Z (eq)	p(unc)	MNI			Brain region
						*x*	*y*	*z*	
Repeated *t*-test on perfusion	Mania < euthymia	6	9.7	3.98	< 0.001	20	−52	38	Right precuneus
		28	8.35	3.77	< 0.001	−28	−52	38	Left superior parietal lobule
		27	6.78	3.48	< 0.001	−8	−74	−45	Left cerebellum lobule VIII-X
		5	6.05	3.31	< 0.001	12	−41	45	Right precuneus
		28	8.35	3.77	< 0.001	−28	−52	38	Left superior parietal lobule
Laterality-by-state	Mania (left vs. right) > Euthymia (left vs. right)	42	4.81	3.77	< 0.001	−20	42	26	Left superior and middle frontal gyrus
		7	4.11	3.38	< 0.001	−35	22	−12	Left anterior insula
	Mania (left vs. right) < euthymia (left vs. right)	27	4.58	3.65	< 0.001	23	44	26	Right superior and middle frontal gyrus
Symptom correlation	Positive correlates to YMRS	40	4.18	3.31	< 0.001	−33	−79	−10	Left occipital fusiform gyrus

YMRS, young mania rating scale; MNI, coordinate system of the Montreal Neurological Institute and Hospital.

The TFCE and ROI approaches did not yield any further significant findings.

### 3.2. Laterality of mania

There were no significant differences in perfusion laterality imbalance, contrasting the mania and euthymic states. At a less stringent threshold (*p* < 0.001, not corrected for multiple comparisons), a trend was observed in that the mania state was associated with a relative imbalance suggesting higher perfusion in the left superior and middle frontal gyrus, see Table 3. Similarly, the right superior and middle frontal gyrus observed a trend toward more significant asymmetry in the euthymia state.

### 3.3. Relation between perfusion and mania ratings

For the entire sample evaluated only in the mania state (*n* = 17), there were no significant correlations to the YMRS scale. At a less stringent threshold (*p* < 0.001, not corrected for multiple comparisons), a trend was observed toward a positive correlation between YMRS ratings and perfusion of the left occipital fusiform gyrus (Table 3).

## 4. Discussion

This study aimed (i) to investigate the differences in brain perfusion between manic and euthymic states in BD-I patients, (ii) to explore potential associations between altered brain perfusion and cognitive status, (iii) and examine the relationship between cerebral perfusion and mania symptom ratings. We detected cognitive impairment during the manic episode, which persisted during the euthymic state at follow-up. However, no significant changes in brain perfusion were observed between the manic and euthymic states. We discuss each of these findings below.

### 4.1. Cognitive function in BD patients during mania

During the manic episode, immediate verbal learning, fluency, and processing speed were found below the normalized values for each subscale. These results agree with those reported in a systematic review, where it was found that during the manic episode, patients showed significant dysfunctions in attention, language, memory, and executive functions (13). However, in the eight subjects who also participated in our follow-up visit, we did not observe any changes in cognitive function between the manic episode and euthymia, suggesting that cognitive function did not improve in euthymia in BD-I (4, 35).

### 4.2. Changes in brain perfusion

There were no significant changes in measured cerebral perfusion between the manic state at follow-up euthymia. Several trends were, however, observed, with reduced perfusion of the right parietal cortex during mania and evidence of more significant left–right perfusion imbalance during mania, particularly in the left superior and middle frontal gyrus. We note that these trends correspond to a general pattern of mania associated with right-hemisphere hypofunction and left-hemisphere hyperfunction (7, 22), *ad hoc* to our hypothesis; yet, these trends should be interpreted cautiously.

We also did not observe significant correlations between the YMRS score and cerebral perfusion in the mania state. A trend toward a negative correlation between YMRS ratings and perfusion of the left occipital fusiform gyrus was observed. Only a few data implicate selective disturbances in the occipital cortex in BD-I, possibly indicating that this trend should be explored in more detail (36, 37).

### 4.3. Cognitive functioning and clinical variables at 6-month follow-up

This study found that, even if the manic symptoms improved, cognitive functioning 6 months later was still impaired. These findings coincide with those found in a prior meta-analysis in which patients with euthymia showed impairment in verbal learning functions and immediate and delayed verbal memory, as well as in tests of executive functions related to problem-solving, verbal interference, and attention change tasks. It should be noted that this systematic review only included cross-sectional studies of patients in different phases of BD. without follow-up (9).

Some factors have been studied to explain the persistent cognitive impairment in BD-I patients, such as the number and severity of episodes, considering chronic patients or patients having a history of multiple episodes suffer from more significant cognitive deficits, age at illness onset, presence of psychotic symptoms, years of stabilization, and pharmacological treatment, since medication may negatively affect cognitive performance (38). However, the population sample we examined had less than 5 years since BD-I diagnosis, with an average of 2.5 years and 1.29 throughout their lifetime; this suggests that cognitive impairment may start early during the disorder. Other studies have found that cognitive impairment may be an endophenotype for BD, as evidence shows that psychomotor speed and response inhibition are observed in unaffected relatives and offspring of BD-I patients (39). Furthermore, some studies have found that cognitive deficits are still evident in euthymic medication-free patients (35). Around two-thirds of BD-I patients experience cognitive problems, directly impacting their ability to function socially and occupationally. Moreover, a pattern of cognitive decline may increase the likelihood of recurring episodes (40).

Open and controlled studies have been made to investigate the outcomes of cognitive rehabilitation interventions for BD patients. Some of these interventions have shown promising results in reducing depressive symptoms and improving executive functions (41). However, more research on cognitive impairments is needed to expand treatment options.

We found no significant correlation between brain perfusion and YMRS score or cognitive performance at baseline or follow-up. The limited sample size might explain this, but in light of recent large-scale, complex phenotypes like mania symptoms or cognition may not lend themselves to simple linear relationships (42).

### 4.4. Clinical significance

A significant decrease in the YMRS scale score and overall clinical improvement was found at follow-up. However, we found no difference in cognitive performance. BD is accompanied by neurotoxic processes that can accelerate the mechanisms of normal aging (43). Neurostructural, alterations in oxidative stress and amyloid metabolism, immune dysregulation, immunosenescence, neurotrophic deficiencies, and telomere shortening have been found in patients with BD-I (44–47). Although these results could be associated with the pharmacological treatment of the patient or with the recovery of global and cognitive functioning after a manic episode, perhaps taking more than 6 months, it is also possible that cognitive alterations are persistent traits, present even without affective symptoms (11, 39, 48, 49).

Our outcomes highlight the relevance of developing new therapeutic strategies aimed at improving and maintaining the cognitive functioning of these patients, as well as possible neuroanatomical targets to direct treatments based on the clinical state of the patients. There is no currently available robust evidence of therapeutic interventions targeting cognitive deficits. Regarding pharmacotherapy, lurasidone, vortioxetine, omega-3 fatty acids, modafinil, vitamin D, and aspirin are currently under investigation in BD-I (3). Functional remediation appears as an excellent option to alleviate psychosocial outcomes in bipolar patients, with an effect that seems to remain in the long term. However, current evidence is insufficient and additional studies are required to prevent neurocognitive impairment and the associated disability in BD patients (50).

### 4.5. Strengths and limitations

Some of the limitations of this study were having a small sample size and the high rate of loss to follow-up (47%). It was impossible to control the pharmacological treatment of patients after the manic episode; this is shown in Supplementary Table 1. Two sets of instruments were used to assess cognitive function among the participants, and scores and cut points were calculated proportionally according to the SCIP-S sub-scores. However, this is one of the few studies that have tracked BD-I patients longitudinally and evaluated brain perfusion and cognitive functioning, which may provide more information about the pathophysiology of cognitive impairment in BD-I.

## 5. Conclusion

This study found limited evidence of alterations in brain perfusion during manic episodes, partly supporting BD-I’s laterality hypothesis. There was evidence of cognitive impairment during mania, and although patients changed to euthymia, their cognitive functioning did not improve after 6 months. Studies in larger populations with extended follow-up periods are needed to explore brain perfusion and cognitive functioning changes in patients with BD-I.

## Data availability statement

The raw data supporting the conclusions of this article will be made available by the authors, without undue reservation.

## Ethics statement

The Instituto Nacional de Psiquiatría Ethics Committee Board reviewed and approved this study. The study was conducted per the Federal legislation and institutional policies. Written informed consent was obtained from participants and family members.

## Author contributions

ME-G, CL, VG, GC-K, KP-B, LM-Q, and MF have been involved in interpreting the results, drafting the manuscript, revising critically for important intellectual content, giving final approval of the version to be published, and agreeing to be accountable for all aspects of the work. CL performed the brain perfusion analysis. All authors contributed to the article and approved the submitted version.

## Funding

This study was supported by the National Institute of Psychiatry “Ramón de la Fuente Muñiz,” CDMX, Mexico.

## Conflict of interest

The authors declare that the research was conducted in the absence of any commercial or financial relationships that could be construed as a potential conflict of interest.

## Publisher’s note

All claims expressed in this article are solely those of the authors and do not necessarily represent those of their affiliated organizations, or those of the publisher, the editors and the reviewers. Any product that may be evaluated in this article, or claim that may be made by its manufacturer, is not guaranteed or endorsed by the publisher.
