# Supplementary material for: Is brain perfusion correlated to switching mood states and cognitive impairment in bipolar disorder type I? A longitudinal study using perfusion imaging approach
Source: Front Psychiatry. 2023 Oct 4;14:1244134. doi: 10.3389/fpsyt.2023.1244134 (PMC10582948; doi:10.3389/fpsyt.2023.1244134)
Supplement: Supplementary file 1 [file Table_1.pdf]

Supplementary material 1. List of patients´ pharmacological treatments at follow-up

	**Treatment**
**Subject 1**	Quetiapine 100mg/ day<br>Magnesium Valproate 800mg/day
**Subject 2**	Haloperidol 7.5mg/day<br>Biperiden 2mg/day<br>Sodium valproate 1000mg/day
**Subject 4**	Quetiapine 225mg/day<br>Magnesium Valproate 800mg /day
**Subject 5**	Magnesium Valproate 800mg/day<br>Lamotrigine 100mg/day
**Subject 7**	Magnesium Valproate 1250mg/day<br>Quetiapine 25mg/day<br>Paroxetine 15mg/day<br>Clonazepam 1mg/day
**Subject 8**	Magnesium Valproate 800mg/day<br>Risperidone 1mg/day
**Subject 9**	Quetiapine 300mg/day<br>Lamotrigine 150mg/day<br>Clonazepam 0.5mg/day
**Subject 10**	Lithium carbonate 900mg/day<br>Olanzapine 10mg/day
